# Childhood microbial experience, immunoregulation, inflammation and adult susceptibility to psychosocial stressors and depression in rich and poor countries

**DOI:** 10.1093/emph/eos005

**Published:** 2012-12-24

**Authors:** Graham A. W. Rook, Charles L. Raison, C. A. Lowry

**Affiliations:** ^1^Department of Infection, Centre for Clinical Microbiology, University College London, London, UK; ^2^Department of Psychiatry, College of Medicine and Norton School of Family and Consumer Sciences, University of Arizona, Tucson, AZ, USA; ^3^Department of Integrative Physiology and Center for Neuroscience, University of Colorado Boulder, Boulder, CO 80309-0354, USA.



As communities undergo the transition from traditional rural life to modern urban lifestyles, the prevalence of chronic inflammatory disorders such as allergies, autoimmunity and inflammatory bowel diseases increases dramatically. It now appears that this is at least in part attributable to diminished efficiency of immunoregulation resulting from inadequate exposure to macro- and microorganisms (Old Friends). These Old Friends had to be tolerated, and they evolved methods of manipulating host immune systems, e.g. priming immunoregulatory pathways, sometimes by secreting molecules that directly expand Treg populations [1]. Thus, they were entrusted by co-evolutionary processes with setting up immunoregulatory circuits (Table 1).

**Table 1.** Major categories of evidence supporting the Old Friends mechanism, fully referenced in [1]

Major categories of evidence
Epidemiological associations
Loss of old friends in urbanized populations
Protection from allergy and juvenile IBD by exposing child <2 years old (or pregnant mother) to farming environment (perinatal exposure)
Protection from allergy by contact with animals, pets and dogs
Less history of allergies in adults with antibodies to Old Friends
Experimental models
Old Friends can block or even treat all chronic inflammatory disorders investigated (allergies, inflammatory bowel disease, autoimmunity)
Clinical observations
Remission of multiple sclerosis following natural helminth infection
Remission of IBD following deliberate self-infection with helminths
Immunoregulatory correlations with skin microbiota and environment
Clinical trials
Helminths for multiple sclerosis
Helminths for IBD (Crohn’s and ulcerative colitis)
Molecular pathways
Cause dendritic cells to drive Treg differentiation
Secrete molecules that cause Treg to proliferate (bacteroides, helminths)
Huge literature on mechanisms of immunoregulation by gut mictobiota

IBD, inflammatory bowel disease; Treg, regulatory T cell.

Recently, these ideas have been expanded to include a subset of depressed patients who demonstrate persistently high levels of inflammatory mediators ‘at rest’ and an exaggerated cytokine response to psychosocial stressors [2]. Such observations prompted the suggestion that some depression in rich urbanized societies is a chronic inflammatory disorder attributable to an immunoregulatory deficit. Several lines of evidence converge to support this possibility. First, prolonged administration of inflammatory mediators such as interferon-α causes a depression-like state that is treatable with selective serotonin reuptake inhibitor antidepressants. Second, the cytokine antagonist infliximab demonstrates antidepressant properties, but only in depressed individuals with evidence of increased peripheral inflammation before treatment [3].

The idea that depression may represent a chronic inflammatory condition in some individuals is increasingly supported by data from developed countries. But we do not yet know whether this association is a universal human attribute or an unintended consequence of modern life. While we do know that chronic inflammatory disorders are less prevalent in traditional rural societies, is this really because contact with the Old Friends has driven efficient regulation of the underlying inflammatory mechanisms? And if so, does this result in less inflammatory response to psychosocial stressors, a lower prevalence of the type of depression that is accompanied by increased biomarkers of inflammation, or both?

Early findings suggested that the answer to these questions should be no, given that cross-sectional studies in developing countries revealed a ‘higher’ prevalence of increased C-reactive protein (CRP) than in USA, indicating that inflammation is more, rather than less, prevalent in such environments [4]. Recently, work by McDade *et al.* [5] has largely resolved this paradox by measuring CRP, interleukin (IL)-6 in longitudinal studies with repeat samples from the same individuals, while documenting the presence of infection at the time of sampling. The results revealed that in Ecuador individuals have peaks of inflammation caused by transient infection that return to very low levels once the infection has resolved [5]. In other words, immunoregulation is efficient, and the inflammatory response is terminated when no longer needed. In contrast, in USA and other developed countries there is often constant low-grade inflammation, manifested as chronically increased CRP or IL-6, in the absence of a normal inflammatory stimulus. Such chronically increased inflammation greatly increases the risk of subsequent inflammatory disease and cardiovascular problems and has been shown in some studies to predict the future development of depression. Thus, major inflammatory episodes appear to be less common in the industrialized world than in developing countries, but despite this inflammatory mechanisms fail to shut down entirely: an immunoregulatory failure.

In McDade’s studies on developing countries, two childhood factors correlated with adult ‘resting’ CRP. First, low birth weight was associated with ‘increased’ adult CRP, consistent with the well-known inverse relationship between birth weight, adult inflammation and risk of cardiovascular disease. But the crucial factor here was the observation that ‘lower’ levels of ‘resting’ CRP in adulthood were associated with ‘higher’ levels of microbial exposure in infancy [6], consistent with the hypothesis that childhood exposure to Old Friends drives effective immunoregulation that persists into adulthood.

This leads our discussion back to psychiatric disorders, bearing in mind that inflammatory mediators can drive depression. What are the consequences for psychiatric disorders of better regulation of background inflammation?

In experimental animals, parental deprivation is a potent inducer of long-term changes to stress responses and immunoregulation. Numerous human studies suggest similar correlations, and another publication by McDade *et al.* confirmed this, but with a fascinating twist. Parental absence in childhood was a significant predictor of increased CRP in adulthood, but ‘only in a subset’ of the cohort [7]. The adults who had a high level of microbial exposure in infancy seemed to be resistant to the long-term proinflammatory effects of this severe childhood stressor [7].

The same was true of perceived stress during the previous month in young adults. CRP correlated with recent perceived stress in the subjects with low microbial exposures in infancy, but not in those with high microbial exposures. Again, exposure to immunoregulation-inducing Old Friends seems to provide resistance to the inflammation-inducing effects of psychosocial stressors [7].

But is this relevant to depression? This brings us to the most recent article from McDade *et al.* [8]. Although depression does exist in their population, they found—in contrast to common observations in the developed world—that it was not associated with increased CRP. This could imply that the association between inflammation and depression is not a universal human occurrence but an unfortunate quirk of our immunodysregulated Western societies. In other words, depression may be increasing in USA not just because life is becoming more stressful, but also because in developed countries our immune systems release more depression-inducing proinflammatory cytokines in response to any given level of psychosocial stressor. The prevalence of depression should therefore be greater in developed countries than in developing ones. Comparative studies are difficult to do, but this is indeed what data collected by the World Health Organization indicate.

To control some of the factors that confound the comparison of depression prevalence rates across highly divergent cultures, we might consider comparing urban and rural populations within rich countries. This has been done repeatedly for other inflammatory conditions. Thus, for example, the protective effects of farming environments against allergies and early-onset inflammatory bowel disease are well documented and require that a child be exposed to that environment during the first 2.5 years of life or during pregnancy [1]. This protective effect is at least partly due to induction of immunoregulation [1]. If similar immunoregulatory mechanisms affect depression, then similar urban–rural differences should exist. A recent meta-analysis found that the prevalence of depression was 39% higher in urban than in rural areas [9]. It is usually assumed that these urban–rural differences are due to greater stressfulness of urban life. For example, a recent functional magnetic resonance imaging study from Germany compared the effects of an experimental social stressor on individuals brought up in urban or rural environments and found striking differences [10]. The authors attributed their findings to different levels of social stressors in individuals with an urban versus rural upbringing. McDade’s work in particular, and an Old Friends perspective more generally, provides an alternative explanation for these findings. While social stressors in young children may differ significantly in these two environments in a wealthy European country such as Germany, it is equally likely that the findings were due to the Old Friends mechanism, with increased downregulation of proinflammatory mediators in subjects with a rural upbringing. The authors of the functional magnetic resonance imaging study did not measure the stress-induced levels of circulating proinflammatory cytokines. The Old Friends view predicts higher levels in the subjects with urban upbringings, and this would be easy to verify.

These concepts are also likely to be important in understanding the health of immigrants. Depression and all the chronic inflammatory disorders discussed here tend to be more common in immigrants than in the birth population from which the immigrant was derived, at least when the migration is from a developing to a developed country. Several findings indicate that it is ‘loss’ of something present in the country of origin rather than exposure to something novel (such as psychosocial stressors) in the destination country that is responsible for the changing disease patterns. For example, disease risk is often greater in second-than in first-generation immigrants. First-generation immigrants to Sweden remain partially protected from ulcerative colitis and Crohn’s disease, perhaps by Old Friends encountered in their countries of origin, but both diseases increase in the second generation. Similarly, migration increases the prevalence of allergies and multiple sclerosis, but the events that modulate risk occur very early in life, usually before migration, with age at migration being crucial. For example, Mexicans, Cubans and African/Caribbean people have a 2- to 3-fold increase in the prevalence of depression if immigration to USA occurs when individuals are less than 13 years old or are born in USA, compared with the prevalence in those who migrate after the age of 13 [11]. Such observations are compatible with the view that adult immunoregulation is modified by childhood exposures to Old Friends and that adult immunoregulation mediates both chronic inflammatory disorders and susceptibility to psychosocial stressors and inflammation-associated depression.

In short, an inflammation-associated form of depression identified in rich countries now appears to be unusual in developing countries and might therefore be an appropriate target for novel immunoregulatory treatments [10].

## FUNDING

C.L.R. receives grant support from the National Center for Complementary and Alternative Medicine (R01AT004698, R01AT007297, R01AT004698-01A1S1) and the Depressive and Bipolar Disorder Alternative Treatment Foundation. He reports the following activities for the previous two years: advisory board participation and related travel funds for Pamlab, Lilly and North American Center for Continuing Education; development and presentation of disease state slides for Pamlab, Pfizer and Johnson & Johnson, as well as related travel funds for these activities; development of continuing medical education material for North American Center for Continuing Education and for CME Incite. C.A.L. was supported by the National Science Foundation under (grant No. 0845550). Any opinions, findings, and conclusions or recommendations expressed in this material are those of the author(s) and do not necessarily reflect the views of the National Science Foundation.

## References

[1] Rook GA (2012). Hygiene hypothesis and autoimmune diseases. Clin Rev Allergy Immunol.

[2] Raison CL, Lowry CA, Rook GAW (2010). Inflammation, sanitation and consternation: loss of contact with
co-evolved, tolerogenic micro-organisms and the pathophysiology and
treatment of major depression. Arch Gen Psychiatry.

[3] Raison CL, Rutherford RE, Woolwine BJ (2012). A randomized controlled trial of the tumor necrosis factor
antagonist infliximab for treatment-resistant depression: the role of
baseline inflammatory biomarkers. Arch Gen Psychiatry.

[4] Gurven M, Kaplan H, Winking J (2008). Aging and inflammation in two epidemiological
worlds. J Gerontol A Biol Sci Med Sci.

[5] McDade TW, Tallman PS, Madimenos FC (2012). Analysis of variability of high sensitivity C-reactive protein in
lowland ecuador reveals no evidence of chronic low-grade
inflammation. Am J Hum Biol.

[6] McDade TW, Rutherford J, Adair L (2010). Early origins of inflammation: microbial exposures in infancy
predict lower levels of C-reactive protein in adulthood. Proc Biol Sci.

[7] McDade TW, Hoke M, Borja JB (2012). Do environments in infancy moderate the association between
stress and inflammation in adulthood? Preliminary evidence from a birth
cohort in the Philippines. Brain Behav Immun.

[8] McDade TW, Borja JB, Adair LS (2012). Depressive symptoms are not associated with inflammation in
younger and older adults in the Philippines. Evol Med Pub Health.

[9] Peen J, Schoevers RA, Beekman AT (2010). The current status of urban-rural differences in psychiatric
disorders. Acta Psychiatr Scand.

[10] Rook GA, Raison CL, Lowry CA (2012). Can we vaccinate against depression?. Drug Discov Today.

[11] Breslau J, Borges G, Hagar Y (2009). Immigration to the USA and risk for mood and anxiety disorders:
variation by origin and age at immigration. Psychol Med.

